# Study of Forms of Compounds of Vanadium and Other Elements in Samples of Pyrometallurgical Enrichment of Ash from Burning Oil Combustion at Thermal Power Plants

**DOI:** 10.3390/ma15238596

**Published:** 2022-12-02

**Authors:** Anton Volkov, Ulyana Kologrieva, Pavel Stulov

**Affiliations:** I. P. Bardin Central Research Institute of Ferrous Metallurgy, 23/9 bdg. 2, Radio Street, 105005 Moscow, Russia

**Keywords:** fly ash, fuel oil, power station, ash recycling, vanadium, soluble forms of vanadium, metal smelting, slag, deposits on the electrode, chemical analysis, XRD, XPS, XRF, MP-AES, titration

## Abstract

The results of the processing of ash from the combustion of fuel oil after roasting with the addition of Na_2_CO_3_ followed by aluminothermic melting are presented. As a result, metallic nickel and vanadium slag were obtained. Studies of slag, metal, and deposits on the electrode were carried out. The resulting metal contains about 90 wt% Ni. The main phases of scurf on the electrode are a solid solution based on periclase (Mg_1–x–y–z_Ni_x_Fe_y_V_z_O), sodium-magnesium vanadate (NaMg_4_(VO_4_)_3_), and substituted forsterite (Mg_2–x–y_Fe_x_Ni_y_SiO_4_). The processing of ash made it possible to significantly increase the concentration of vanadium and convert it into more soluble compounds. Vanadium amount increased from 16.2 in ash to 41.4–48.1 V_2_O_5_ wt% in slag. The solubility of vanadium was studied during aqueous leaching and in solutions of H_2_SO_4_ and Na_2_CO_3_. The highest solubility of vanadium was seen in H_2_SO_4_ solutions. The degree of extraction of vanadium into the solution during sulfuric acid leaching of ash was 18.9%. In slag, this figure increased to 72.3–96.2%. In the ash sample, vanadium was found in the form of V^5+^, V^4+^ compounds, vanadium oxides VO_2_ (V^4+^), V_2_O_5_ (V^5+^), and V_6_O_13_, and nickel orthovanadate Ni_3_(VO_4_)_2_ (V^5+^) was found in it. In the slag sample, vanadium was in the form of compounds V^5+^, V^4+^, V^3+^, and V^(0÷3)+^; V^5+^ was presented in the form of compounds vanadate NaMg_4_(VO_4_)_3_, NaVO_3_, and Ca_x_Mg_y_Na_z_(VO_4_)_6_; V^3+^ was present in spinel (FeV_2_O_4_) and substituted karelianite (V_2–x–y–z_Fe_x_Al_y_Cr_z_O_3_). In the obtained slag samples, soluble forms of vanadium are due to the presence of sodium metavanadate (NaVO_3_), a phase with the structure of granate Ca_x_Mg_y_Na_z_(VO_4_)_6_ and (possibly) substituted karelianite (V_2–x–y–z_Fe_x_Al_y_Cr_z_O_3_). In addition, spinel phases of the MgAl_2_O_4_ type beta-alumina (NaAl_11_O_17_), nepheline (Na_4–x_K_x_Al_4_Si_4_O_16_), and lepidocrocite (FeOOH) were found in the slag samples.

## 1. Introduction

Vanadium is one of rare metals, and it has a rather high cost. The main consumer of vanadium is ferrous metallurgy, where it is used in the form of ferrovanadium as an alloying element [1] For example, in Russia, about 78% of the consumed vanadium goes into ferrous metallurgy [2]. In addition, vanadium finds application in the composition of catalysts; today, energy-storage devices based on vanadium salts are believed to be promising.

The main raw materials for vanadium are titanomagnetite ores, where vanadium is found as an impurity in iron and titanium minerals [1,3]. Usually, a concentrate is obtained from them, which is reduced to pig iron in a blast furnace. Vanadium converter slag is obtained from this cast iron in an oxygen converter, which is a product for hydrometallurgical processing [4,5]. The production is characterized by a large amount of waste; it does not provide for complex processing of slag [6].

Some of the sources of vanadium are oils, natural bitumen, and asphaltites. The highest concentrations of vanadium are typical for high-sulfur and heavy oils, high-boiling fractions of oil, and products of its processing (fuel oil, tar, coke). Reserves of heavy, high-viscosity oil account for about 80% of the world’s oil resources [7]. According to Yaschenko et al., the world’s potential resources of vanadium in heavy oil and natural bitumen are about 125 million tons [8]. Oil raw material accounts for about 8% of the Canadian production [7]. The amount of vanadium in oil from different fields varies greatly. According to Ismail et al., the content of vanadium in oil from different countries is (ppm): Morocco—0.6; Kuwait—22.5; Syria—15.0; Yemen—0.4–4.1; West Texas, 7.9; East Texas—1.2 [9].

During oil refining, vanadium is concentrated in its high-boiling fractions. The content of vanadium in petroleum coke ranges from 350 to 2500 ppm [10] and in heavy fuel oil up to 5000 ppm. Quite a great deal of vanadium is contained in waste products (fly ash, slag, bottom ash) formed during the combustion of heavy oil fractions. Slags and sludges are considered poor if they contain up to 10% pentoxide and rich if they contain more than 10% vanadium pentoxide [11]. The amount of vanadium in the ash depends on the chemical composition of the feedstock, combustion efficiency, the amount and composition of additives to eliminate deposits, neutralize acids, and reduce corrosion. Thus, fly ash from Al-Aqaba power station (Jordan) contains 3.1% V [12]; the North American three-pass fire tube package boiler, from 0.5 to 5 wt% V [13]; the power station in Jordan, 0.02 wt% V [14]; a fuel oil power plant and oil refinery in Cuba, 0.48 wt% V [15]; the Damascus thermal power station, 5.0 wt% V [16]; the USA, 0.45–6.00 wt% [17]; and the bottom ashes from an electric oil fuel power plant located in Southern Italy, 0.39 wt% V [18]. The amount of vanadium pentoxide in the ash of heavy fuel oil in Venezuela reaches 75%, in the countries of the Middle East 8%, in Oklahoma 22%, and in Texas 1.4% [19]. The composition of the sludge from washings of the equipment of gas-oil thermal power plants includes from 5 to 9 wt% of vanadium compounds in terms of V_2_O_5_ [20]. Enriched ash from fuel oil combustion at Berezovskaya GRES (Belarus) contains 2.94 wt% V and sludge 1.21 wt% V [21]. The ash from the GRES in Konakovo (Russia) contains 7.82 wt% V_2_O_5_ [22]. Moreover, 22.65 wt% V_2_O_5_ is contained in the ash residue of the Baku power plant (Azerbaijan) [23]. The amount of vanadium in the ash from the combustion of fuel oil at the Kirov CHP (Apatity, Murmansk region) is 9.67 wt% V_2_O_5_ [24]. In addition, the chemical composition of the ash depends on the place of deposition in the furnace [18] as well as on the distance of the ash layer from the tubes of the boiler system [25]. The amount of vanadium near the tube reaches 80%, while in the outer layer, it is about 20% [25].

The high content of vanadium exacerbates the corrosion of oil pipelines, boiler equipment, oil refineries, and storage tanks [19]. In the process of oil refining, vanadium can be incorporated into environmentally harmful emissions; along with other metals, it leads to the deactivation of catalysts [7]. The work [26] shows the effect of vanadium on the combustion quality of fuel oils. Given this as well as the high cost and rarity of vanadium, the task of extracting vanadium from ash-processing products is relevant.

In many countries, the industrial production of vanadium from petroleum raw materials has not been mastered. Vanadium is obtained from oil in countries such as the USA, Canada, and Japan [8]. The review [27] systematized the characteristics and applications of heavy fuel oil ash. In addition to extracting metals [28], ash can be used to produce activated carbon and carbon nanotubes, remove pollutants from the environment, and produce polymer composites and building materials [27].

The hydrometallurgical methods of ash processing described in the literature are divided into acidic [29,30,31], alkaline [16,32,33,34], and aqueous [10,22,23,27,35]. Beforehand, the material can be roasted using additives. At the same time, vanadium and nickel are usually separated by precipitation and extraction. The proposed pyrometallurgical processing methods imply the production of ferrovanadium with an admixture of nickel [36,37]. Pyrometallurgical methods of processing various wastes containing Ni and V are known, as a result of which it was possible to isolate Ni into a separate commercial product (ferronickel), while V remained in the slag [38]. This method of processing seems to be the most promising in connection with a more complete separation of target metals and the possibility of obtaining highly liquid commercial products directly from waste. Furthermore, after pyrometallurgical processing, waste (slags) can be a promising raw material for the extraction of vanadium. Therefore, this method was taken as a basis for processing ash from the burning of fuel oil at thermal power plants.

This paper presents the results of studying the forms of compounds of vanadium and other elements in samples of pyrometallurgical enrichment of ash from fuel oil combustion at thermal power plants. The proposed method of enrichment with the production of rich vanadium slag can become a method for processing ash from the combustion of heavy oil fractions. However, it was first necessary to evaluate the technological properties of such slag and compare them with the properties of the original ash.

## 2. Materials and Methods

### 2.1. Materials

The studies were carried out on a sample of ash from fuel oil combustion used by the company SIA “FerroLat” (Liepaja, Latvia) in the production process. For melting and lining the furnace, we used periclase (magnesite) brick grade P91-No. 1 according to GOST 4689-94 produced by OgneuporEnergoHolding LLC and caustic magnesite powder grade PMK-75 produced by Stromeks LLC, aluminum grit grade KP (Al ≥ 98.0 wt%) according to TU 1791-99-023−99 produced by PKF TSVET LLC3.

Anhydrous lithium nitrate (99% purity, Alfa Aesar, Haverhill, MA, USA) and fused lithium borates Li_2_B_4_O_7_ and LiBO_2_ (99.98% purity, Fluxana GmbH & Co. KG, Bedburg-Hau, Germany) were used as a flux in X-ray fluorescence analysis.

Concentrated hydrochloric HCl (grade “chemically pure”), nitric HNO_3_ (grade “pure for analysis”), and hydrofluoric HF (grade “chemically pure”) acids were used to transfer the samples to an aqueous solution.

### 2.2. Methods

#### 2.2.1. Ash Recycling—Metal Smelting

The ash sample was preliminarily dried at 105 °C in a drying drum. Then, sodium carbonate Na_2_CO_3_ (soda) was added to the dried sample, and the mixture was stirred and placed in a muffle furnace. The firing was carried out at a temperature of 800 °C for 2 h. Experimental melting was carried out in an electric arc furnace DP-0.1. Characteristics of the electric furnace are as follows: maximum voltage up to −140 W; maximum current—2500 A; maximum operating temperature—2250 °C; type of heaters—carbon electrodes—75 mm; distance between electrodes 180 mm; the size of the working chamber (length-width-height)—460 × 460 × 475 mm; overall dimensions of the furnace (length-width-height)—862 × 850 × 835 mm. The furnace is equipped with a power voltage cut-off system in case of overloads. The lining of the furnace walls is three-layered: the inner layer is made of magnesite bricks, the middle layer is made of fireclay, and the outer one is asbestos. The hearth is made up of a coal block. The roof of the furnace is lined with magnesite bricks. Burnt ash, aluminum metal, and steel scrap are used as charge materials.

**Figure 1:**
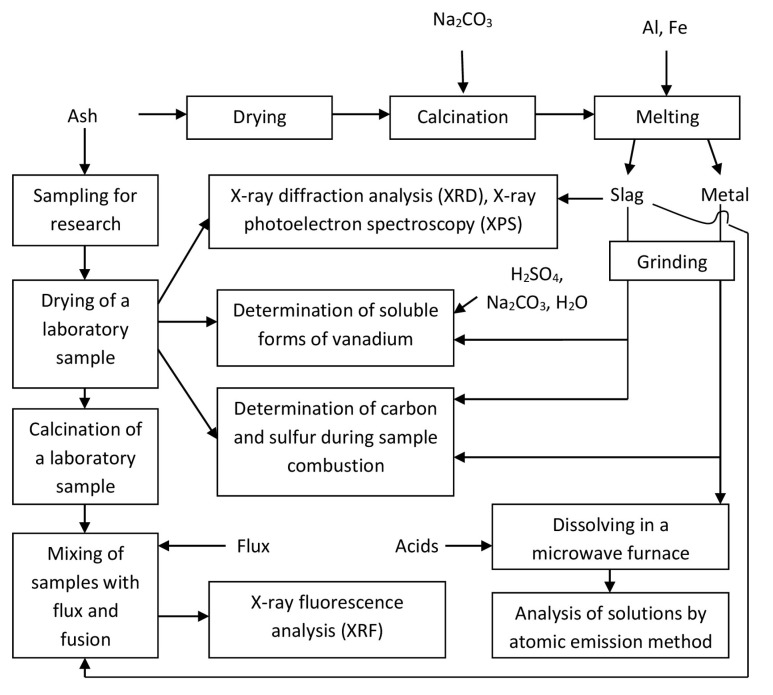
The scheme of processing and research of ash and its products.

The scheme of experiments with ash is shown in Figure 1.

#### 2.2.2. Chemical Analysis

Samples of ash from fuel oil combustion were preliminarily dried for 2 h at a temperature of 110 °C. Carbon and sulfur in dried samples were determined using a CS-2000 analyzer (Eltra, Haan, Germany). Loss on ignition was determined in a muffle furnace when heated to 1050 °C until the mass change ceased.

Ash and slag samples were chemically analyzed using an AXIOS^max^ Advanced X-ray fluorescence spectrometer (PANalytical, Almelo, The Netherlands). Samples were prepared using an Eagon 2 sample melting furnace (PANalytical, Almelo, The Netherlands).

Synthetic standard samples were used for calibration since there are no standard samples of the chemical composition of vanadium ash. To do this, a mixture of oxides of the elements that make up the ash in various proportions was prepared. Preliminarily, calcined reagents of chemical grade and analytical grade were used. Pre-calcined reagents of the “hc” and “chda” brands were used. Fluxes (lithium tetraborate, lithium nitrate, and lithium carbonate) for fusion were dried for 3 h, then mixed in a certain proportion [39] and stored in a desiccator. When melting calcined samples that did not contain carbon, a flux was used, which was a mixture of 65 wt% Li_2_B_4_O_7_ and 35 wt% LiBO_2_.

Metal samples formed during the reduction melting of burnt ash were analyzed by microwave plasma atomic emission spectrometry (AES-MEP) on an Agilent 4200 MP spectrometer (Agilent, Santa Clara, CA, USA). Ash and metal samples were dissolved using a MARS-6 microwave system (CEM Corporation, Matthews, NC, USA). To do this, 3 mL of concentrated hydrofluoric acid, 3 mL of concentrated nitric acid, and 6 mL of concentrated hydrochloric acid were added to 0.2 g of the sample. The resulting mixture was kept in closed vessels at 210 °C for 2 h, while the pressure in the vessels reached 30 atm. After that, 30 mL of a 4% boric acid solution was added to neutralize the remaining hydrofluoric acid. It was heated for 15 min to a temperature in the vessel of 170 °C, kept at this temperature for 10 min, and then was cooled for 15 min.

#### 2.2.3. X-ray Diffraction Analysis

X-ray phase analysis was performed using an ARL 9900 Workstation (Thermo Fisher Scientific, Waltham, MA, USA). The samples were pressed in the form of a powder on a boric acid substrate. The measurements were carried out in vacuum (up to 4 Pa). Qualitative analysis was carried out using the ICDD PDF-4 database and the Crystallographica Search-Match software package (ICDD, PA, USA). The program PhaseQuantX was used for quantitative X-ray phase analysis.

#### 2.2.4. Determination of Soluble Forms of Vanadium

##### Determination of Sulfuric Acid-Soluble Vanadium

A slag sample weighing 20 g was placed in a glass beaker with a capacity of 400 mL. Then, 200 mL of dilute sulfuric acid (7% H_2_SO_4_ aqueous solution) was poured into the beaker. The solution was stirred using an overhead stirrer. 

The filter cake was washed with 50 mL of water. A slag sample weighing 20 g was placed in a glass beaker with a capacity of 400 mL, and 200 mL of dilute sulfuric acid (7% H_2_SO_4_ aqueous solution) was poured into the beaker. The solution was stirred using an overhead stirrer. After 30 min of leaching, the solution was filtered through a paper filter into a Bunsen flask under vacuum. The filter cake was washed with 50 mL of water. Then, the resulting solution was poured from the flask into a measuring cylinder to accurately determine the volume of the resulting filtrate. Three 10 mL aliquots of the filtrate were pipetted into 250 mL flasks. Then, 10 mL of concentrated sulfuric acid and 30 mL of hot water were added to these flasks. A solution with a concentration of 1.5 g/L KMnO_4_ was added dropwise until a stable pink color appeared. One minute later, one drop of a 50 g/L KNO_2_ solution was added to the solution (until the pink color disappeared). Excess NO_2_-ions were neutralized after a couple of minutes by adding 10 mL of a 5% solution of (NH_2_)_2_CO. As an indicator, 5 drops of a 0.2% solution of phenylanthranilic acid were added. The resulting solution, while stirring with a magnetic stirrer, was titrated with a solution of 0.1 N (NH_4_)_2_Fe(SO_4_)_2_ (salt solution in an aqueous solution of 1:7 H_2_SO_4_) until the color of the solution changed from violet to green.

The content of the soluble form of vanadium in terms of pentoxide C(V_2_O_5_) was calculated by an equation:

C(V_2_O_5_) = C_t_∙V_t_∙M(V_2_O_5_)/m∙100% (1)

where C_t_—solution concentration (NH_4_)_2_Fe(SO_4_)_2_, mol-eq/L; V_t_—solution volume (NH_4_)_2_Fe(SO_4_)_2_, which was for titration, L; M(V_2_O_5_)—molar mass of the equivalent V_2_O_5_, g/ mol-eq; m—mass of the slag sample involved in titration, g.

##### Determination of Sodium Carbonate-Soluble Vanadium

A slag sample weighing 20 g was placed in a glass beaker with a capacity of 400 mL. Then, 200 mL of a hot solution of 50 g/L Na_2_CO_3_ was poured into a beaker. The glass was placed on a heating plate, and the mixing of the solution was carried out using an overhead stirrer. After 30 min of leaching at 80 °C, the solution was filtered through a paper filter into a Bunsen flask under vacuum. The filter cake was washed with 50 mL of water. All subsequent actions are the same as in the determination of acid-soluble vanadium.

The content of the sodium carbonate-soluble form of vanadium in terms of pentoxide was calculated by an Equation (1).

##### Determination of Water-Soluble Vanadium

In this case, the course of research is the same as in the determination of soda-soluble vanadium, and only hot water was used instead of a Na_2_CO_3_ solution.

Calculation of the content of water-soluble forms of vanadium was calculated by an Equation (1).

#### 2.2.5. X-ray Photoelectron Spectrometry

To determine the forms of compounds of elements, the method of X-ray photoelectron spectrometry (XPS) was used using a PHI 5500 ESCA spectrometer (Physical Electronics, Chanhassen, MN, USA). Photoemission was excited using nonmonochromatic AlKα radiation from a standard source with a power of 300 W. The analysis area diameter was 1.1 mm. Approximation was performed by a non-linear least squares method using Gauss–Lorentz functions.

## 3. Results and Discussion

The starting material is a wet black powder with a specific smell of gasoline. The weight of the sample in air decreases, possibly due to the evaporation of the light fraction of hydrocarbons. Chemical analysis was carried out on a pre-dried sample. The sample was dried for 2 h at 110 °C. When calcined at 1050 °C for 2 h, the sample melts and partially evaporates. As a result, a molten mass of red-brown color remains. When the sample is heated for 2 h at 500 °C, the sample does not melt, and a brown powder remains. In the sample calcined at this temperature, the sulfur content decreases by 3–4 times (0.64–1.24%). Sample humidity (110 °C) is 44%. Loss on ignition (1050 °C) is 71%.

The chemical composition of dried and calcined ash is presented in Table 1. The main components of the ash are compounds of vanadium, iron, and nickel. For comparison, Table 1 presents the ranges of element contents in ash according to our previous work [40]. These data are based on the analysis of many ash samples.

In previous works, we have shown the influence of the degree of oxidation and forms of compounds of elements on the manufacturability of processing the corresponding raw materials [41,42,43]. To study the mineral composition and the degree of oxidation of elements in vanadium raw materials, the following methods are described: X-ray phase analysis [44,45,46], X-ray photoelectron spectrometry [45,47,48], titrimetric [44,45,49], electrochemical methods [44], mass spectral [50], thermal analysis [48], infrared spectrometry [48], electron microscopy [46,47], study of the fine structure of the X-ray absorption spectrum (XAFS) [17], and nuclear magnetic resonance method [27].

In our previous work [39], the results of the study of an ash sample by XRD and XPS methods are presented. The studied ash sample contained FeSO4 sulfate and vanadium in the form of V^5+^ and V^4+^ compounds adsorbed hydrocarbons C_n_H_m_, i.e., carbon in the form of organic compounds with O–C and O=C bonds. X-ray phase analysis revealed the following compounds in the ash: FeNi_2_O_4_, VO_2_, Ni_3_(VO_4_)_2_, V_2_O_5_, and V_6_O_13_. Table 2 compares the data of works by different authors. Our data [40] are consistent and complement the data of other authors [12,13,14,18,22,23,24,27]; in particular, the presence of vanadium in different oxidation states was confirmed.

Figure 1 shows the scheme of ash processing. The research and development of the relevant technology is the subject of a separate article, so the relevant data are not presented here. As a result of melting, a metal was obtained, consisting mainly of nickel and vanadium slag. Chemical composition of the metal is as follows (%wt): 0.06 C; 0.49 Al; 0.09 Si; 0.07 P; 0.9 S; 0.25 V; 2.26 Cr; 0.03 Mn; 3.45 Fe; 0.05 Co; 89.9 Ni; and 0.24 Cu. The resulting slag was heterogeneous, so several samples were studied. The chemical composition of the slag is shown in the Table 3. The content of vanadium in terms of its oxide V_2_O_5_ in slag samples ranges from 41 to 48 wt%. A high carbon content was found in slag sample no. 3. This may be due to the ingress of particles from the furnace electrode. A sample of deposits on the furnace electrode was studied separately. They are a white solid.

**Figure 2:**
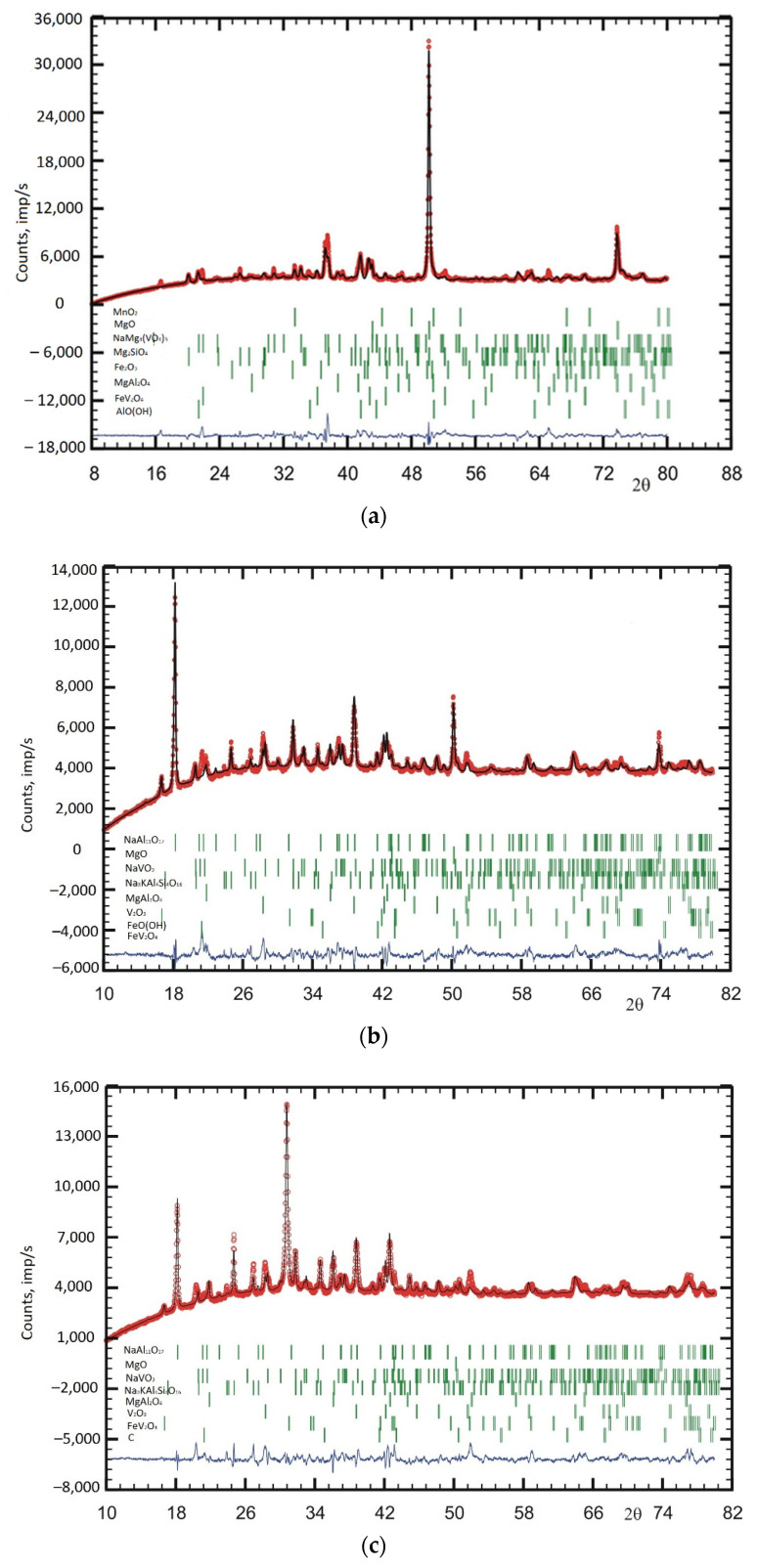

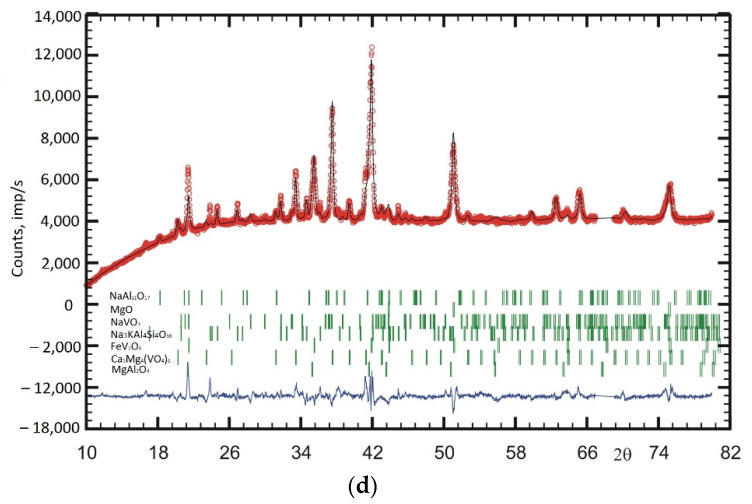
Experimental (red), theoretical (black), and differential (blue) diffractograms acquired from deposits on the electrode (**a**) and slag samples #1 (**b**), #2 (**c**), and #3 (**d**). Green dashes correspond to the positions of reference diffraction peaks of correspondent phases.

The studied samples are complex mixtures in terms of phase composition (Figure 2, Table 4). The main phase in the deposit sample on the electrode is a solid solution based on periclase (Mg_1–x–y–z_Ni_x_Fe_y_V_z_O).

However, judging by the unit cell parameter (4.2149(3) Å compared to 4.2112 Å for pure MgO), the degree of substitution is low: at the level of a few atomic percent. Sodium-magnesium vanadate NaMg_4_(VO_4_)_3_ and forsterite (substituted) Mg_2–x–y_Fe_x_Ni_y_SiO_4_ are also present in significant concentrations. As impurities in the mixture, two phases with a spinel structure were found (one, with a small parameter of 8.1335(11) Å, probably contains aluminum as a B-cation, and the second, with an average parameter of 8.3395(15) Å, is probably enriched in Fe^3+^ and V^3+^ in the B sublattice): hematite Fe_2_O_3_ and (presumably) pyrolusite MnO_2_. The study of weak maxima also suggests the presence of the AlOOH diaspore in the mixture (Figure 2a, Table 4). However, the fact of the existence of hydroxide in the heat treatment product is not fully explained, and the presence of this phase should be confirmed additionally.

The phase composition of slag naturally changes along with the change in the chemical composition. In slag sample no. 1, the main phases are beta-alumina NaAl_11_O_17_, periclase (MgO, a = 4.2110(3) Å), sodium metavanadate NaVO_3_, nepheline Na_4-x_K_x_Al_4_Si_4_O_16_, and substituted karelianite V_2–x–y–z_Fe_x_Al_y_Cr_z_O_3_ (Figure 2b). There are also two phases with spinel structures: one enriched in Al (α = 8.1597(11) Å) and one enriched in transition metals (α = 8.363(3) Å). The reflection around 16.66 2θ was attributed to lepidocrocite FeOOH. However, as in the case of soot, the presence of hydroxide requires further confirmation.

The phase composition of slag sample no. 2 (Figure 2c, Table 4) is very similar to that of slag no. 1 (Figure 2b, Table 4). Sample no. 2 contains a significant amount of graphite. In slag sample no. 3 (Figure 2d, Table 4), due to the higher content of calcium oxide and the lower content of aluminum and sodium oxides, beta-alumina and NaVO_3_ are impurities, while the main phases are periclase, a phase with a spinel structure (possibly MgV_2-x_Al_x_O_4_—α = 8.2818(11) Å, nepheline, and the phase with the garnet structure Ca_x_Mg_y_Na_z_(VO_4_)_6_. The second phase with the spinel structure (α = 8.3387(15) Å) is also present as an impurity.

Table 5 presents the results of determining the soluble forms of vanadium in the original ash and in the slag obtained from it. The highest solubility of vanadium is observed in sulfuric acid solutions.

Table 6 shows the degree of extraction of vanadium into solution during sulfuric acid leaching. In the same place for comparison is the content of vanadium-containing phases.

As can be seen from the data presented in Table 5 and Table 6, ash processing significantly increased the concentration of vanadium and converted it into more soluble compounds. The vanadium content in terms of V_2_O_5_ increased from 16.2 wt% in the range of 41.4–48.1 wt% in the slag (see Table 6). The degree of vanadium extraction into the solution during sulfuric acid leaching of ash was 18.9%. In slag, this indicator increased to 72.3–96.2%. The three slag samples differ in chemical and phase composition (Table 3 and Table 4). When comparing the data in Table 4, Table 5 and Table 6, it can be noted that the degree of vanadium extraction into the slag solution is the lowest in a sample with a higher content of spinel (FeV_2_O_4_), which is consistent with the data of [42,43,51,52,53]. With an increase in the content of the phases of sodium metavanadate (NaVO_3_) and karelianite (V_2–x–y–z_Fe_x_Al_y_Cr_z_O_3_), the content of soluble forms of vanadium increases.

According Zhang et al., oxides V_2_O_5_ and V_3_O_16_ in vanadium converter slag after roasting with CaO are poor soluble in (NH_4_)_2_CO_3_ solutions [54], and vanadium is deposited in the form of vanadate NaV_3_O_8_·H_2_O [55]. The presented phases have a different composition and structure, which leads to a difference in solubility. Sample slag no. 3 with the lowest content of soluble forms of vanadium contains the granate (Ca_5_Mg_4_(VO4)_6_) phase. At the same time, garnet phases (Ca_3_Fe_3.5_V_1.5_O_12_) are formed during firing of vanadium converter slag with limestone [43] and should be characterized by good solubility. However, other calcium vanadates Ca_3_V_2_O_8_, Ca_10_V_6_O_25_, and Ca_2_V_2_O_7_ are also characteristic of similar samples [54]. A detailed study of the process of extracting vanadium from slag is the subject of our further research.

**Figure 3:**
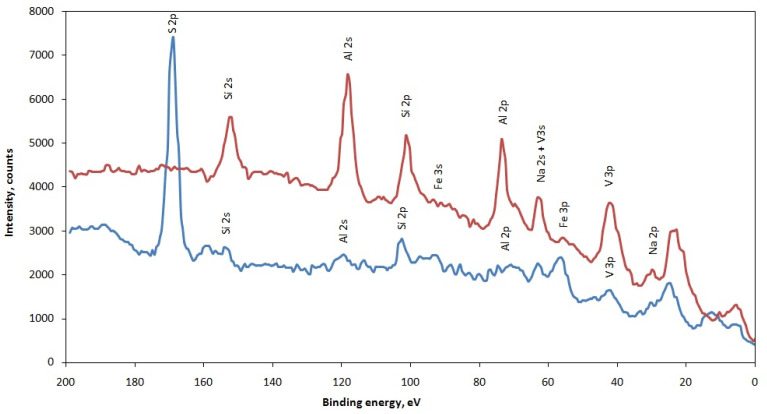
XPS spectra from the surface of ash (blue) and slag no. 3 (red) samples.

Figure 3 demonstrate the results of XPS analysis of ash and slag no.3 samples. According to this spectrum, a change in the chemical composition and forms of elements in the ash sample after its processing is detected.

**Figure 4:**
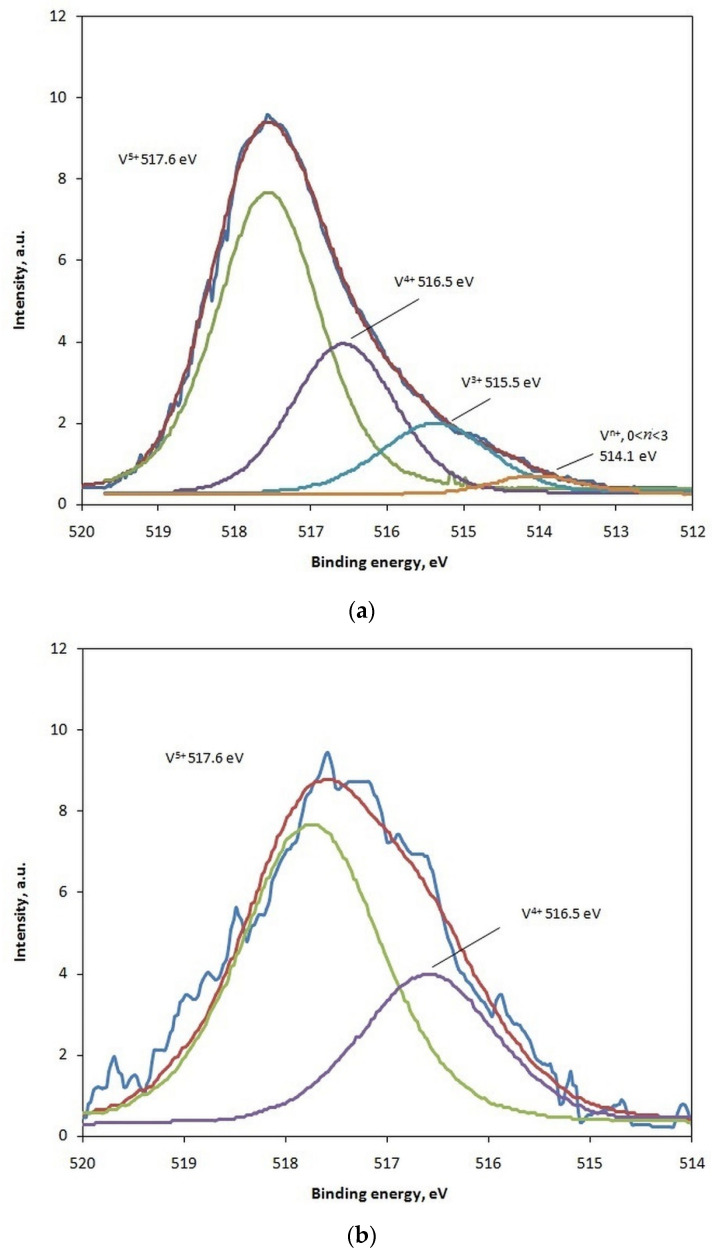
XPS V2p spectra from the surface of slag no. 3 (**a**) and ash (**b**) samples.

The V2p3 spectra are shown in Figure 4. Mathematical decomposition of the spectrum made it possible to reveal two lines in the ash (Figure 4b) and four lines in the slag (Figure 4a). The results of calculations of the content of vanadium in different oxidation states are presented in Table 7. The results of XPS are consistent with the data of X-ray phase analysis.

Vanadium oxides V^4+^O_2_, V^5+^_2_O_5_, V_6_O_13_, and nickel orthovanadate Ni_3_(V^5+^O_4_)_2_ were found in the initial ash sample. Oxide V_6_O_13_ is intermediate between oxides V_2_O_5_ and VO_2_. Thus, in the initial ash sample, vanadium is in the form of V^5+^ and V^4+^ compounds. According to XPS data, vanadium is present in the slag sample in the form of V^5+^, V^4+^, V^3+^, and V^(0÷3)+^ compounds. X-ray phase analysis revealed V^5+^ in the form of vanadate NaMg_4_(VO_4_)_3_, NaVO_3_, and Ca_5_Mg_4_(VO_4_)_6_ and V^3+^ in spinel (FeV_2_O_4_) and karelianite (V_2_O_3_). Separate phases of vanadium with oxidation states V^4+^ and V^(0÷3)+^ were not detected by X-ray phase analysis. Based on this, it can be assumed that they are present in the composition of the amorphous phase, the content of which in slag samples varies greatly from 4 to 33 wt%. Given the above, we can assume the following chain of chemical transformations during the recovery of ash:

V_2_O_5_; (VO_4_)^3−^ → V_n_O_2n+1_ (V_6_O_13_) → VO_2_ → V_3_O_5_; V_n_O_2n-1_ (4 ≤ *n* ≤ 8) → V_2_O_3_; (V_2_O_4_)^2−^ → V^(0÷3)+^ (2)

The sulfur line S2p (a doublet of peaks 2p3/2–2p1/2) is located in the region of binding energies corresponding to the sulfate ion SO_4_^2−^. The position of the line S 2p3/2 169.1 eV coincides with the binding energy of sulfur in iron sulfate FeSO_4_. No sulfur lines were found in the slag sample.

**Figure 5:**
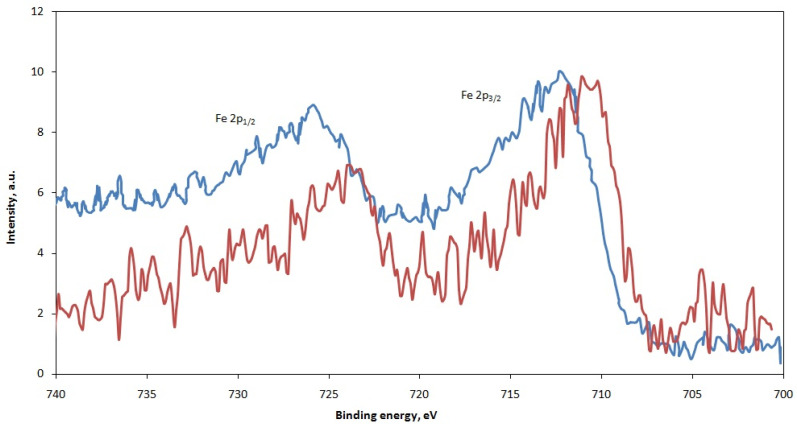
XPS Fe2p spectra from the surface of slag #3 (red) and ash (blue) samples.

The spectrum of the ash sample contains the Fe 2p3/2 line with an energy of about 712 eV, which is also characteristic of iron sulfate FeSO_4_ (Figure 5). In the slag sample, the Fe 2p3/2 line is located at about 710 eV, which corresponds to the binding energy in a compound of the Fe_3_O_4_ type. According to X-ray phase analysis (see Table 4), no independent phase of magnetite was found; however, spinel phases enriched in transition metals are observed. Based on these data, it can be assumed that the following chemical reaction occurs during roasting and ash reduction:

3FeSO_4_ + C = Fe_3_O_4_ + 3SO_2_↑ + CO_2_↑ (3)

## 4. Conclusions

As a result of roasting ash with the addition of Na_2_CO_3_ followed by aluminothermic melting, a slag containing up to 48 wt% V_2_O_5_ was obtained. The resulting vanadium slag is superior in vanadium content to both ash and converter slag, which is currently the main raw material for the production of vanadium and its compounds. Thus, this slag is a promising raw material for existing titanomagnetite processing plants to produce V_2_O_5_. Data on the content of soluble forms of vanadium in the resulting slag showed its advantage over the original ash. This allows us to look optimistically at the possibility of further processing of slag. It can be used both directly for the production of vanadium alloys and for the production of vanadium compounds, including high-purity pentoxide, by hydrometallurgical processing. In addition, during the processing of ash, nickel is separated into a separate metal product, which can be used, for example, to produce stainless steel. This will make it possible to receive additional profit in the processing of ash as well as to reduce the consumption of reagents for its processing, reducing the amount of waste.

## Figures and Tables

**Table 1:** Chemical composition of ash samples from fuel oil combustion, wt%.

Component	Data for Different Samples	Content (Our Data)	
	Range of Contents (after Calcination)	The Original Sample	After Calcination
Na_2_O	0.10–4.74	0.13	0.45
MgO	0.09–2.14	0.08	0.26
Al_2_O_3_	0.61–7.10	0.41	1.40
SiO_2_	0.11–23.00	0.07	0.23
P_2_O_5_	0.14–0.53	0.04	0.15
S	0.39–11.21	2.8	9.67
Cl	0.13–0.23	n/d *	0.07
K_2_O	0.02–2.04	0.005	0.02
CaO	0.08–4.94	0.05	0.18
TiO_2_	0.11–0.75	0.03	0.11
V_2_O_5_	15.18–70.70	16.2	55.9
MnO	0.04–0.42	0.03	0.09
Fe_2_O_3_	7.15–64.91	2.7	9.3
Co_3_O_4_	0.06–4.60	0.02	0.06
NiO	1.30–18.59	4.0	13.8
ZnO	0.21–1.99	0.08	0.33
SrO	0.03–0.06	n/d *	0.007
MoO_3_	0.06–0.75	0.06	0.24
BaO	0.94–1.64	n/d *	n/d
PbO	0.04–0.11	0.03	0.11
Cr_2_O_3_	0.17–0.51	n/d *	n/d
C	0.16–91.1 **	65.0	0.01
Moisture	0.15–49.0 **	44.0	~0
Ash content	0.19–97.0 **	29.0	~100

* n/d, not detected; ** the results are given for a non-calcined sample.

**Table 2:** Forms of compounds of elements in ash.

Element	Compound Forms (Our Data [40])	Compound Forms (Data from Other Authors [12,13,14,18,22,23,24,27]
Vanadium	VO_2_, V_2_O_5_, V_6_O_13_, Ni_3_(VO_4_)_2_, vanadium in the form of compounds V^5+^, V^4+^	V^5+^, V^4+^, V^3+^—sulfate VOSO_4_·H_2_O, oxides V_2_O_3_, V_2_O_5_, Mg_3_V_2_O_8_, VO_2_·V_2_O_3_, spinel FeO·V_2_O_3_, and vanadium bronze NaV_6_O_15_
Nickel	FeNi_2_O_4_, Ni_3_(VO_4_)_2_	spinel NiFe_2_O_4_, sulfate NiSO_4_, oxide NiO, sulfide Ni_3_S_2_
Iron	FeNi_2_O_4_, FeSO_4_	oxides Fe_3_O_4_, Fe_2_O_3_, hydroxide FeOOH (goethite), sulfates FeSO_4_, Fe_2_(SO_4_)_3_, FeH(SO_4_)_2_·nH_2_O (rhomboclase), sulfide
Sulfur	FeSO_4_	metal sulfates (CaSO_4_, NiSO_4_, VOSO_4_·H_2_O, Fe_2_(SO_4_)_3_, CuSO_4_·H_2_O, Al_2_(SO_4_)_3_·17H_2_O, ZnSO_4_ and PbSO_4_), sulfite CaSO_3_, sulfides, elemental sulfur, and thiophene.
Silicon and aluminum		quartz SiO_2_, aluminosilicates (gelenit Ca_2_Al[(Si,Al)_2_O_7_], anorthite Ca[(Al_2_Si_2_)O_8_], kaolinite H_4_Al_2_Si_2_O_9_, mullit, zeolites and cancrinite), sulfate–alunogen Al_2_(SO_4_)_3_·17H_2_O; potassium hydrosilicate KHSi_2_O_5_
Other	hydrocarbons C_n_H_m_, carbon in the form of organic compounds with bonds O–C, O=C.	carbon and organic compounds, metal oxides, arsenates

**Table 3:** Chemical composition of slag and deposits on the electrode, %wt.

Sample	Deposits on the Electrode	Slag No. 1	Slag No. 2	Slag No. 3
Al_2_O_3_	7.9	23.8	27.3	14.7
CaO	1.94	3.03	1.83	6.99
Cr_2_O_3_	0.52	0.5	0.52	0.71
Fe	5	3.8	4.5	15.1
MgO	49	5.6	2.8	5.9
MnO	0.15	0.11	0.07	0.16
P_2_O_5_	0.039	0.032	0.041	0.027
S	0.03	0.08	0.04	0.02
SiO_2_	4.04	3.78	6.77	4.18
TiO_2_	0.13	0.22	0.18	0.18
V_2_O_5_	23.9	48.1	41.4	42.6
Na_2_O	3.9	8.1	6.5	3.2
K_2_O	n/d *	0.12	0.14	n/d *
NiO	2.6	2.4	2.3	4.9
CuO	0.05	0.03	0.04	0.03
ZnO	0.09	0.05	0.03	0.05
Ga_2_O_3_	n/d *	0.02	0.03	0.03
SrO	0.01	0.02	0.01	0.02
ZrO_2_	0.012	0.008	0.006	0.005
MoO_3_	0.01	0.02	0.01	0.01
C	0.18	0.46	6.08	0.09

* n/d, not detected.

**Table 4:** The quantitative phase composition of slag and deposits on the electrode, %wt.

Phases	Deposits on the Electrode	Slag No. 1	Slag No. 2	Slag No. 3
Periclase (MgO)	43.9	13.2	1.1	16.2
Sodium-magnesium vanadate (NaMg_4_(VO_4_)_3_)	13.1	n/d *	n/d *	n/d *
Forsterite (Mg_2_SiO_4_)	16.8	n/d *	n/d *	n/d *
Spinel 1 (rich in Al–MgAl_2_O_4_)	6.0	5.2	8.0	5.7
Spinel 2 (rich in Fe и V–FeV_2_O_4_)	3.9	1.2	0.5	35.3
Hematite (Fe_2_O_3_)	2.4	n/d *	n/d *	n/d *
Pyrolusite (MnO_2_)	1.4	n/d *	n/d *	n/d *
Diaspore (AlOOH)	2.7	n/d *	n/d *	n/d *
Beta-alumina (NaAl_11_O_17_)	n/d *	17.7	8.6	0.43
Sodium metavanadate (NaVO_3_)	n/d *	13.5	8.8	4.2
Nepheline (Na_3_KAl_4_Si_4_O_16_)	n/d *	8.2	13.0	9.9
Karelianite (V_2_O_3_)	n/d *	9.2	7.5	n/d *
Lepidocrocite (FeOOH)	n/d *	1.36	2.7	n/d *
Graphite 2H (C)	n/d *	n/d *	6.8	n/d *
Granate (Ca_5_Mg_4_(VO_4_)_6_)	n/d *	n/d *	n/d *	26.1
Amorphous phase	10	30	33	2

* n/d, not detected.

**Table 5:** Content of soluble forms of vanadium in slag samples, %wt.

Sample	V_2_O_5_ (Total)	V_2_O_5_ (Sulfuric Acid-Soluble)	V_2_O_5_ (Sodium Carbonate-Soluble)	V_2_O_5_ (Water-Soluble)
Ash	16.2	3.06	-	-
Slag No. 1	48.1	46.3	30.7	17.6
Slag No. 2	41.4	39.0	20.7	24.4
Slag No. 3	42.6	30.8	7.6	4.4

**Table 6:** The degree of vanadium extraction into the solution, % rel.

Sample	V_2_O_5_ (Total), wt%	Degree of Vanadium Extraction, % rel.	Spinel (FeV_2_O_4_),wt%	Sodium Metavanadate (NaVO_3_), wt%	Karelianite (V_2_O_3_), wt%	Granate (Ca_5_Mg_4_(VO_4_)_6_), wt%
Ash	16.2	18.9	n/d *	n/d *	n/d *	n/d *
Slag No. 1	48.1	96.2	1.2	13.5	9.2	n/d *
Slag No. 2	41.4	94.2	0.5	8.8	7.5	n/d *
Slag No. 3	42.6	72.3	35.3	4.2	n/d	26.1

* n/d, not detected.

**Table 7:** Content of vanadium with different oxidation degrees in ash and slag samples.

Sample	Binding Energy, eV	Oxidation Degree	Content, % at.
Ash	516.5	V^4+^	33
	517.6	V^5+^	67
Slag No. 3	514.1	V*^n+^*, 0 < *n* < 3	4
	515.5	V^3+^	16
	516.7	V^4+^	30
	517.4	V^5+^	50

## Data Availability

Data are contained within the article.
